# Noninvasive prenatal screening for patients with high body mass index: Evaluating the impact of a customized whole genome sequencing workflow on sensitivity and residual risk

**DOI:** 10.1002/pd.5603

**Published:** 2019-12-20

**Authors:** Dale Muzzey, James D. Goldberg, Carrie Haverty

**Affiliations:** ^1^ Myriad Women's Health South San Francisco CA USA; ^2^ Myriad Genetics Salt Lake City UT USA

## Abstract

**Objective:**

Women with high body mass index (BMI) tend to have reduced fetal fraction (FF) during cell‐free DNA‐based noninvasive prenatal screening (NIPS), causing test failure rates up to 24.3% and prompting guidelines that recommend aneuploidy screening other than NIPS for patients with significant obesity. Because alternatives to NIPS are only preferable if they perform better, we compared the respective sensitivities at different BMI levels of traditional aneuploidy screening and a customized whole‐genome sequencing NIPS.

**Method:**

The relationship between FF, aneuploidy, and BMI was quantified from 58 105 patients screened with a customized NIPS that does not fail samples because of low FF alone. Expected analytical sensitivity as a function of aneuploidy and BMI (eg, trisomy 18 sensitivity when BMI = 35) was determined by scaling the BMI‐ and aneuploidy‐specific FF distribution by the FF‐ and aneuploidy‐specific sensitivity calculated from empirically informed simulations.

**Results:**

Across all classes of obesity and assuming zero FF‐related test failures, analytical sensitivity for the investigated NIPS exceeded that of traditional aneuploidy screening for trisomies 13, 18, and 21.

**Conclusion:**

Relative to traditional aneuploidy screening, a customized NIPS with high accuracy at low FF and a low test‐failure rate is a superior screening option for women with high BMI.

## Abstract 2

What's already known about this topic?

- Women with high body mass index (BMI) often receive a test failure on noninvasive prenatal screening (NIPS) because of low fetal fraction (FF).
- The American College of Medical Genetics and Genomics recommends offering traditional aneuploidy screening to patients with “significant obesity.”
- NIPS offerings differ in their efficacy at low FF.

What does this study add?

- Irrespective of BMI and without FF‐based test failures, it is possible for a customized NIPS to provide all women with accurate prenatal screening.

**What's already known about this topic?**

- Women with high body mass index (BMI) often receive a test failure on noninvasive prenatal screening (NIPS) because of low fetal fraction (FF).
- The American College of Medical Genetics and Genomics recommends offering traditional aneuploidy screening to patients with “significant obesity.”
- NIPS offerings differ in their efficacy at low FF.

**What does this study add?**

- Irrespective of BMI and without FF‐based test failures, it is possible for a customized NIPS to provide all women with accurate prenatal screening.

## 1 INTRODUCTION

Each year in the United States, millions of pregnant women undergo screening to detect fetal aneuploidy on chromosomes 13, 18, and 21. Depending on the tested population, the reported collective incidence of fetal trisomy on these chromosomes ranges from approximately 1 in 100 (1,2) to 1 in 250.^1^ While some women opt for definitive diagnosis via chorionic villus sampling (CVS) or amniocentesis (these procedures are the recommended follow‐up for all screening modalities^2^), these invasive procedures carry a risk of pregnancy loss (medical societies report an estimated rate of 0.1%‐0.3%^3^), and in the case of amniocentesis, patients are tested relatively late in pregnancy (typically 15‐ to 20‐week gestation). Therefore, there is clinical utility and patient desire for noninvasive screening modalities to identify pregnancies at increased risk for aneuploidy at an earlier gestational age with high sensitivity and specificity.

Two prenatal screening approaches are widely used today. The first relies upon measurements that do not involve DNA (“non‐DNA screening”), including serum marker levels (eg, concentrations of alpha‐fetoprotein and pregnancy‐associated placental protein A), and imaging analysis (eg, nuchal translucency) collected in the first and/or second trimester. The second approach is noninvasive prenatal screening (NIPS) via cell‐free DNA (cfDNA).^4^

Non‐DNA screening indirectly tests for trisomy 21 (T21), trisomy 18 (T18), and trisomy 13 (T13) by measuring biomolecule concentrations and ultrasound features that differ in affected and normal pregnancies. There are many permutations of non‐DNA screening (eg, combined screening, sequential screening, and integrated screening), with integrated screening showing the highest sensitivity. Though seminal studies (eg, the FaSTER^5^ Trial) have characterized performance of non‐DNA screening for trisomy 21, herein we use Baer et al.^1^ as our reference for non‐DNA screening performance because it reports sensitivity results for T13, T18, and T21, plus it involved greater than 10x more patients than the FaSTER Trial, greater than 90% of whom received integrated screening. Non‐DNA screening sensitivity for T21 and T18 was 92.9% and 93.2%, respectively,^1^ but together with the specificities (96.0% for T21, 99.6% for T18, calculated from Tables 2 and 3 in^1^), the positive predictive values (PPV) for these trisomies are lackluster: 6.2% for T21 and 14.8% for T18 (calculated from Tables 2 and 3 in^1^). For confirmed T13 cases, non‐DNA screening returned abnormal results in 80.4% of patients,^1^ making this number the effective sensitivity; however, because non‐DNA screening does not specifically identify T13 as the source of abnormality, specificity and PPV cannot be directly calculated.

NIPS directly interrogates cfDNA extracted from maternal plasma, which consists primarily of maternal‐derived DNA but critically also contains a minority of genomic material from the pregnancy. Relative to serum‐ and imaging‐based approaches, NIPS has higher sensitivity, specificity, and PPV (99.7%, 99.96%, and 96.7%, respectively, for T21^6^). The sensitivity of NIPS is not constant for all pregnancies, rather, the ability to detect aneuploidy scales with the proportional share of fetal‐derived cfDNA in the maternal plasma (ie, the “fetal fraction” or “FF”).^7^ Many NIPS laboratories fail samples below a FF threshold because of concerns about reporting false negatives as a result of diminished sensitivity.^8^, ^9^, ^10^ However, low‐FF performance is both platform and laboratory dependent: Modeled versions of the two common NIPS platforms—the whole‐genome sequencing (WGS) and single‐nucleotide polymorphism (SNP) methods—show that WGS has higher sensitivity for low‐FF samples at a fixed specificity level.^7^ The many laboratories offering NIPS via WGS have implemented the molecular and computational aspects of the methodology differently, meaning that performance may vary.

Factors that affect FF include gestational age, chromosome abnormalities, and body mass index (BMI). FF rises with gestational age likely because of increased placental size, but the effect is relatively subtle, with FF increasing 0.1% per week on average between weeks 10 and 21 of gestation.^11^ Chromosome abnormalities affect the size and structure of the placenta: T13 and T18 pregnancies tend to have compromised placentas and low FF; T21 pregnancies, by contrast, have mildly elevated FF.^12^ High BMI is associated with lower FF, potentially because of higher turnover of maternal adipose tissue^13^ or because maternal tissue is relatively more abundant than placental tissue.

Patients with high BMI have an elevated test‐failure rate—as high as 24.3% in obese women^14^—on NIPS platforms with a FF threshold.^13^, ^14^, ^15^ Reports of this elevated test‐failure rate prompted the American College of Medical Genetics and Genomics (ACMG) to recommend against using NIPS in patients with “significant obesity.”^16^ Despite stating that NIPS is “the most sensitive screening option,” ACMG instead recommended that such patients receive “aneuploidy screening other than NIPS,” such as non‐DNA screening.^16^ Other medical societies have not provided specific guidance about patients with high BMI. Because “significant obesity” is not well defined, this recommendation potentially means that many US patients—greater than 25% with at least class I obesity and greater than 10% with at least class II obesity^14^, ^17^, ^18^—would be treated differently based on their height and weight alone. Further, adherence to this recommendation could create inequity in patient care because of ethnicity‐specific differences in the distribution of BMI.^19^

Here, we explore NIPS performance in patients with high BMI using an NIPS methodology that does not impose an FF failure threshold. Our guiding premise was that not performing NIPS on women with high BMI is only justified if the expected NIPS sensitivity actually drops below the sensitivity of non‐DNA screening. In a cohort of geater than 58 000 NIPS patients, we elucidated the relationship between FF, BMI, and aneuploidy. By combining these data with an empirically informed adaptation of our model of WGS sensitivity, we calculated the expected NIPS sensitivities for T13, T18, and T21 for different BMI classes, and we compared these values to sensitivities previously reported for non‐DNA screening.

## 2 METHODS

### 2.1 Patient cohort

The study included 58 105 patients who underwent WGS‐based NIPS over an 8‐month period with the Prequel Prenatal Screen (Myriad Women's Health, South San Francisco, California) and whose height, weight, and ethnicity were reported on the test requisition form. Patients from New York State or who opted out of research were excluded from the study. The protocol was reviewed and designated as exempt by Western Institutional Review Board because it involved de‐identified patients who had consented to anonymized research, and it complied with the Health Insurance Portability and Accountability Act (HIPAA).

### 2.2 BMI calculation and NIPS results

**Figure 1:**
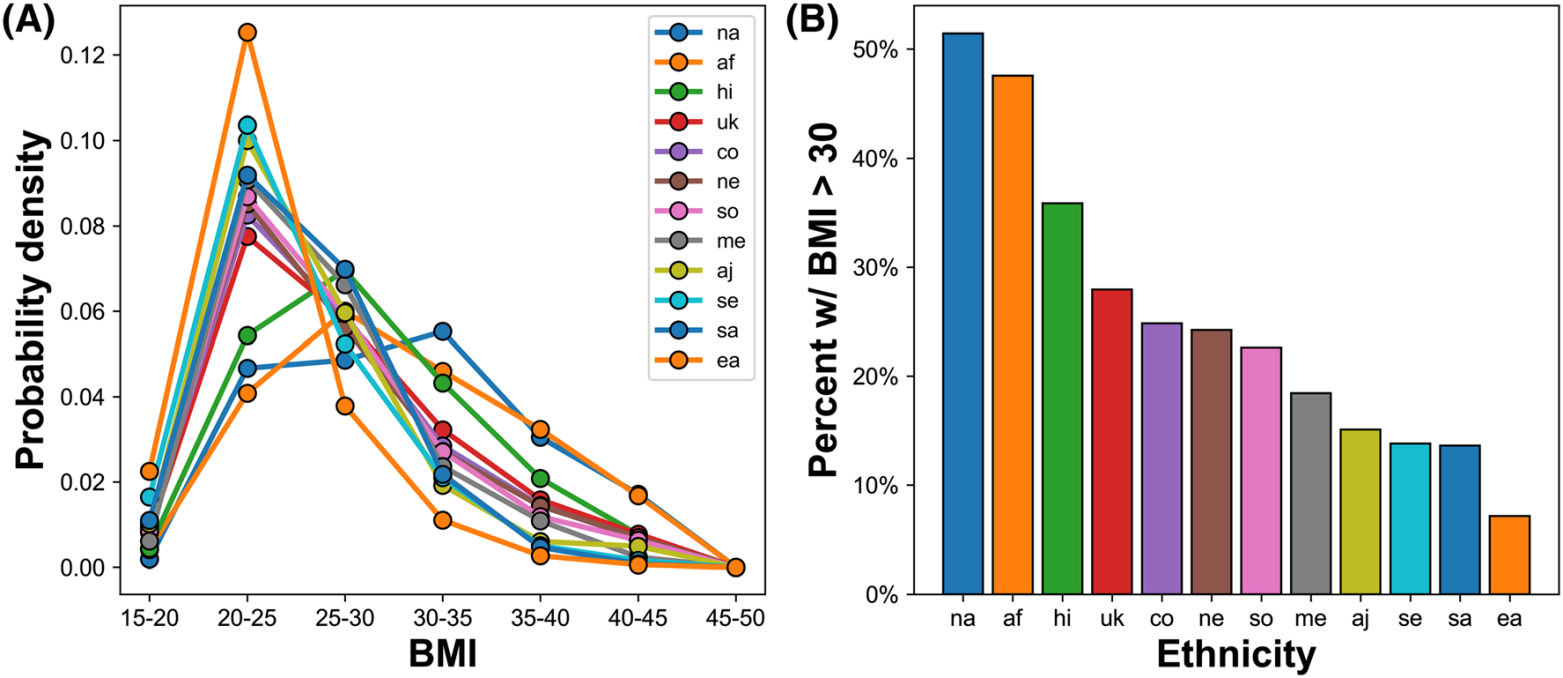
Ethnicity‐specific bias in body mass index (BMI) among pregnant women undergoing noninvasive prenatal screening (NIPS). A, For ethnicities in the NIPS cohort with greater than or equal to 100 patients, normalized histograms binned by BMI class are indicated. B, For each ethnicity in (A), the percent of patients with BMI > 30 is shown. af: African. aj: Ashkenazi Jewish. co: Caucasian/Other. ea: East Asian. hi: Hispanic. me: Middle Eastern. na: Native American. ne: Northern European. sa: South Asian. se: Southeast Asian. so: Southern European. uk: unknown [Colour figure can be viewed at http://wileyonlinelibrary.com]

BMI for patients in the cohort was calculated from maternal height (m) and weight (kg) as (weight/height^2^).^20^ For most analyses, BMI was evaluated in steps of five, corresponding to established classes: BMI < 25 is “normal,” 25 ≤ BMI < 30 is “overweight,” 30 ≤ BMI < 35 is “class I obese,” 35 ≤ BMI < 40 is “class II obese,” and BMI ≥ 40 is “class III obese”.^20^ Figure 1 shows the frequency of different BMI levels as a function of ethnicity, revealing multiple ethnicities in which at least one in four women is obese and underscoring the importance of characterizing aneuploidy screening performance in this population.

Aneuploidy was detected via a *z* score that measures deflections in a chromosome's WGS read‐depth relative to a disomic expectation.^21^, ^22^, ^23^ For instance, a sample was called positive for T21 if the median depth among equally sized bins tiling chromosome 21 had a sufficiently high *z* score relative to the corresponding medians of euploid samples. Fetal fraction was inferred using a regression model that calculates a weighted sum of the normalized read depth in bins tiling autosomes.^24^

### 2.3 Calculation of BMI‐ and aneuploidy‐dependent analytical sensitivity

**Figure 2:**
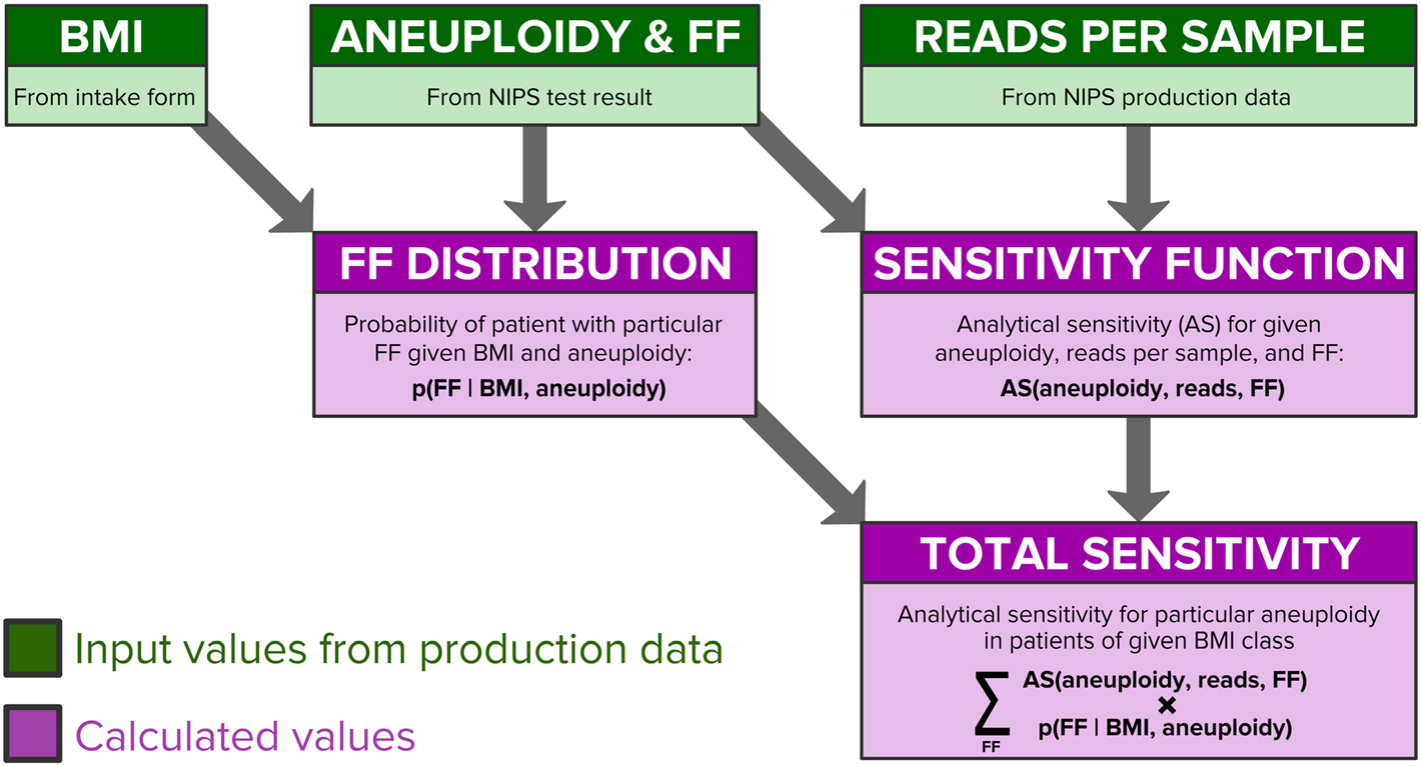
Workflow depicting how a range of observed and calculated values yielded a BMI‐ and chromosome‐specific estimate of analytical sensitivity. Input values are shown in green. Maternal body mass index (BMI) was calculated from weight and height measurements provided on the test order. FF, ploidy status, and reads per sample were measured from NGS data gathered during noninvasive prenatal screening (NIPS) (reads per sample were downward adjusted to model Poisson statistics, as described in Section 2). Calculated values and descriptions are indicated in purple and described in Section 2 [Colour figure can be viewed at http://wileyonlinelibrary.com]

For a given BMI class and aneuploidy, the expected analytical sensitivity (Figure 2, “Total Sensitivity” box) was calculated by weighing the aneuploidy‐ and FF‐specific analytical sensitivity (Figure 2, “Sensitivity Function” box; see “Empirically informed WGS simulation to measure sensitivity as a function of FF” in the Supporting Information Data S1) by the probability of observing a pregnancy with particular FF, aneuploidy, and BMI levels (Figure 2, “FF distribution” box; see “Fetal‐fraction distributions as a function of BMI and aneuploidy” in Supporting Information Data S1). Inputs to this analysis are shown in green boxes in Figure 2. This weighted product was evaluated at FF levels between 0% and 4% (the FF probability distribution was normalized over this range to sum to 100%) in increments of 0.1%, and the weighted values were summed to yield an expected analytical sensitivity for the entire low‐FF range. To estimate the sensitivity for all samples in a particular BMI class—not just restricting to those with low FF—the sum of weighted products was evaluated from 0% to 40% FF (the FF probability distribution was re‐normalized to sum to 100% over this larger range). The following equation describes the calculation of analytical sensitivity (“*AS*”) as a function of FF, number of reads, aneuploidy, and BMI.

$$\sum_{\mathrm{FF}}\mathrm{AS}\left(\text{aneuploidy},\text{reads},\mathrm{FF}\right)*p\left(\mathrm{FF}|\mathrm{BMI},\text{aneuploidy}\right)$$

## 3 RESULTS

### 3.1 Why a modeling approach was needed

Our goal was to measure NIPS sensitivity for T13, T18, and T21 in particular BMI classes and at different FF levels. The ideal, but unfortunately impractical, way to make such a measurement would be to count how frequently NIPS correctly identified aneuploid pregnancies at given BMI and FF: This approach is untenable because while high BMI is rather common, low FF is quite rare, and aneuploidy itself is very rare, together making observed cases too infrequent to power confident measurements, even with a very large data set. For instance, suppose the population frequencies for class III BMI, FF < 2%, and aneuploidy were 10%, 2%, and 1%, respectively, in order to have 100 aneuploid samples from which to calculate sensitivity, the cohort would need 5 000 000 patients (100/[0.1 * 0.02 * 0.01]) just for this one slice of the population of interest.

Instead, we used the modeling approach outlined in Figure 2 and described in Section 2. Clinical data (BMI), NIPS screening results (FF and aneuploidy calls), and assay quality‐control data (reads per sample) for 58 105 patients screened sequentially were analyzed to predict (a) how the FF distribution shifts in response to BMI and particular aneuploidies and (b) how analytical sensitivity levels change as a function of aneuploidy, FF, and reads per sample (see Section 2 and Supporting Information Data S1). Resolving both the FF distribution and the screen's sensitivity as a function of FF enabled calculation of expected analytical sensitivity for a given aneuploidy in a BMI class by summing over all FF values in a range of interest (see Section 2 and Supporting Information Data S1).

### 3.2 Determining how BMI and aneuploidy affect FF distribution

**Figure 3:**
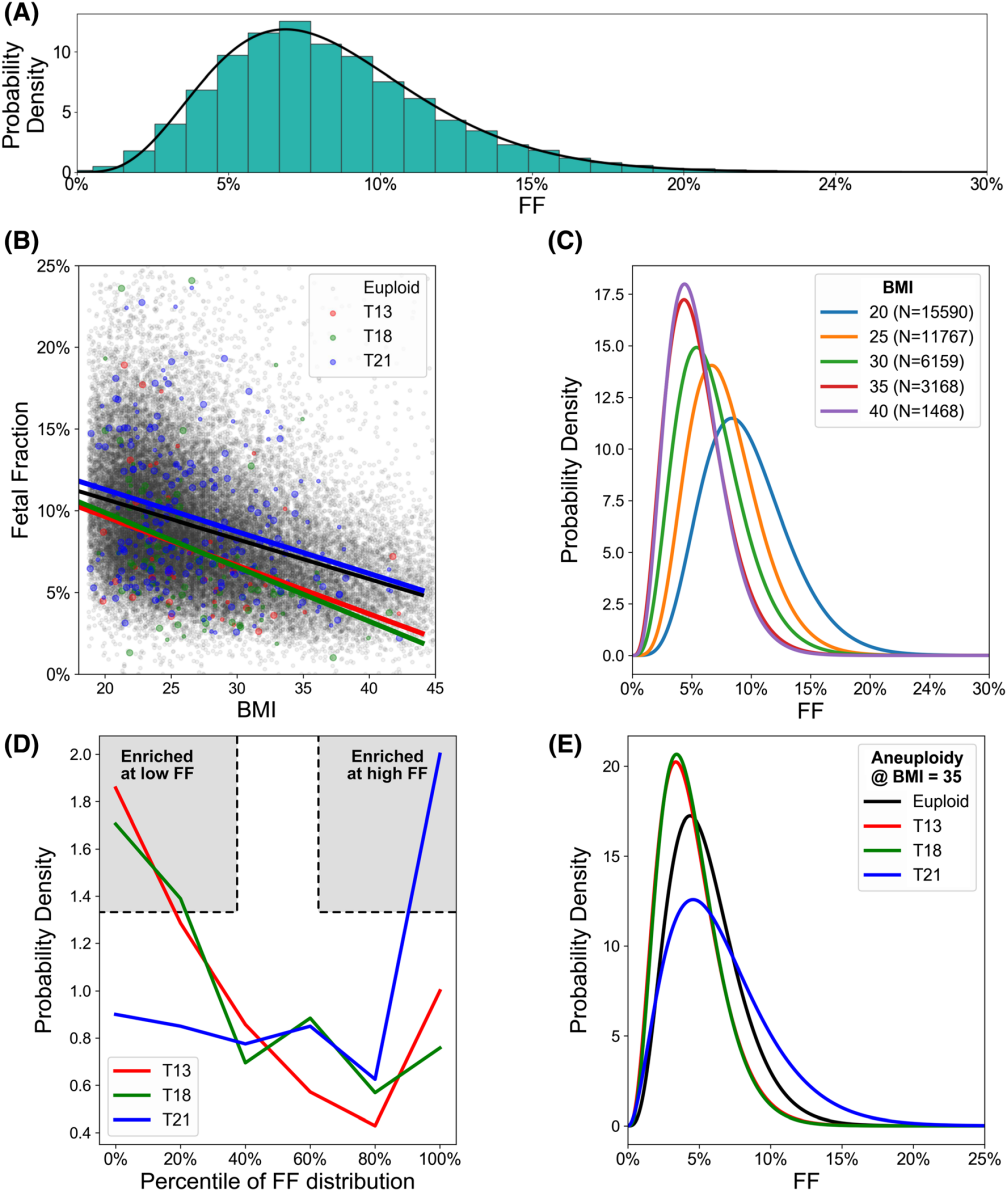
Exploring and quantifying how fetal fraction (FF) scales with body mass index (BMI) and fetal ploidy. A, The empirical FF distribution (teal bars) is well approximated by a beta distribution (black line). B, FF is plotted as a function of maternal BMI and shaded based on being euploid (gray) or screen‐positive for T13, T18, or T21. Large colored dots are those with confirmed clinical outcome gathered via laboratory‐driven recontact of clinics with screen‐positive patients. Fit lines represent the linear best fit to the data with corresponding color. The slope and intercept (m, b) of aneuploidies were Negatives, (‐0.0024, 0.156); T13, (‐0.0030, 0.156); T18, (‐0.0033, 0.165); T21, (‐0.0025, 0.164). On average, FF decreases as BMI increases, and T13 and T18 pregnancies tend to have lower FF than euploid and T21 pregnancies. C, FF frequency distribution as a function of BMI and irrespective of fetal ploidy. The number of patients in each BMI class is indicated in the legend. Each trace is a beta distribution fit to the empirical data. D, Each trace depicts the probability density of the percentile of screen‐positive aneuploid samples relative to euploid samples in the same BMI class (see Section 2). Because they have higher probability at low percentiles, T13 and T18 tend to have lower FF than euploid samples; by contrast, T21 positives tend to have higher FF than euploid samples because the trace is elevated at high percentiles. E, The BMI‐specific inferred FF distributions for each ploidy state can be deduced from data in panels A to D; the particular traces are shown for a BMI of 35 [Colour figure can be viewed at http://wileyonlinelibrary.com]

Though it had previously been observed that BMI and aneuploidy affect FF levels, our modeling approach required detailed resolution of a quantitative relationship among these factors. The relative frequencies of different FF levels are well described by a beta distribution (Figure 3A); thus, we sought to determine how the shape of the beta distribution would change for different chromosomal aneuploidies and BMI classes. First, we observed via linear regression analysis of the raw data (Figure 3B) that FF tends to fall as BMI increases (downward slope of the linear fit in Figure 3B and leftward shift of the entire distribution in Figure 3C). Next, we found that T13 and T18 have downward‐shifted FF (ie, their FF levels were at low percentile values relative to the distribution of euploid samples; Figure 3B,D), and T21 had upward‐shifted FF, with values comparable with the high percentile range of euploid samples. By combining these observations as described in the Section 2 and Supporting Information Data S1, we approximated the beta distributions of FF for each aneuploidy and BMI class; sample distributions for a BMI of 35 are shown in Figure 3E (distributions for other BMI levels shown in Figure S1).

### 3.3 Empirically informed simulation of WGS sensitivity for common aneuploidies

**Figure 4:**
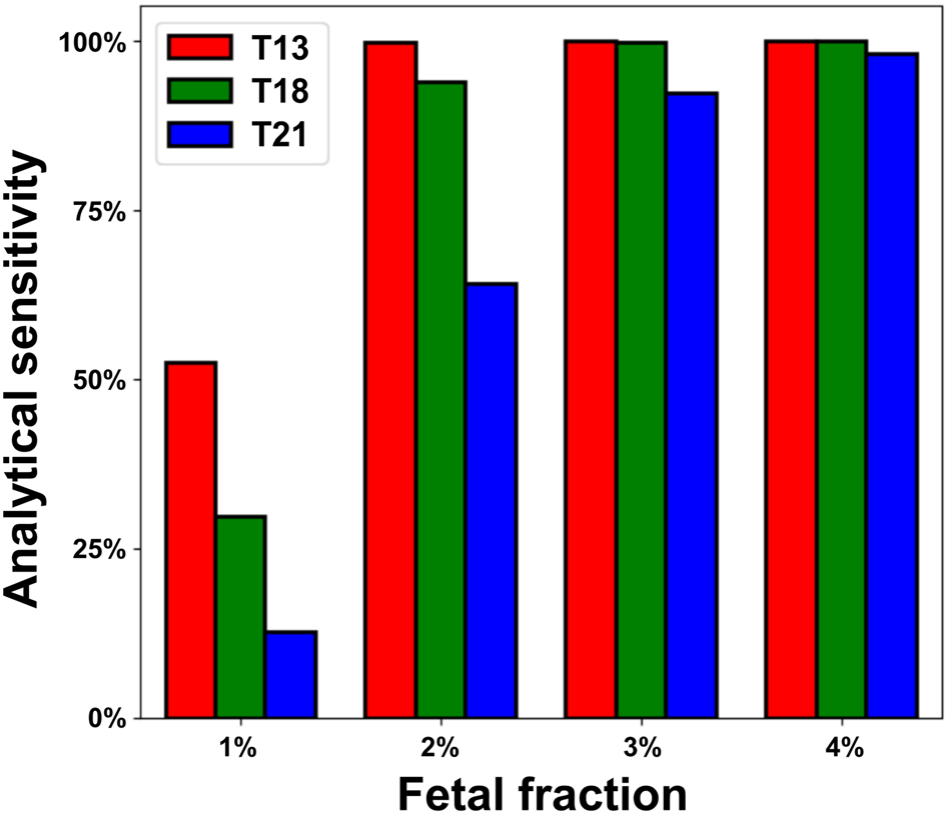
Simulated analytical sensitivity as a function of fetal fraction (FF) and fetal aneuploidy. For the whole‐genome sequencing (WGS) method of noninvasive prenatal screening (NIPS), sensitivity varies by chromosome in a size‐dependent manner [Colour figure can be viewed at http://wileyonlinelibrary.com]

Previous comparison of the WGS and SNP methodologies modeled the performance of each platform in idealized conditions,^7^ but our aim in this study was to describe empirical sensitivity of our customized WGS methodology. By analyzing data from sequential clinical samples processed in our laboratory (see Supporting Information Data S1), we determined the number of reads per sample at which our WGS‐based NIPS behaves in a Poisson manner (Figure S2) and then used this reads‐per‐sample level in our WGS simulations^7^ to predict analytical sensitivity for T13, T18, and T21 at low FF (Figure 4). As anticipated,^25^ these empirically informed sensitivity estimates were comparable with the idealized levels from our previous analysis.^7^

The correspondence between Figure 3D and Figure 4 has two noteworthy features that enable high sensitivity for our implementation of the WGS method of NIPS. First, even though FF levels of T13 and T18 were downward shifted (Figure 3D), sensitivity was relatively high on these chromosomes (as compared with T21) because of their larger size (Figure 4). Second, despite T21 having lower sensitivity relative to T13 and T18 at low FF levels because of its size, T21 pregnancies tend to have upward‐shifted FF levels, meaning that WGS‐based NIPS is sensitive in the FF regime where it is needed.

### 3.4 Expected WGS‐based NIPS analytical sensitivity exceeds that of non‐DNA screening

**Figure 5:**
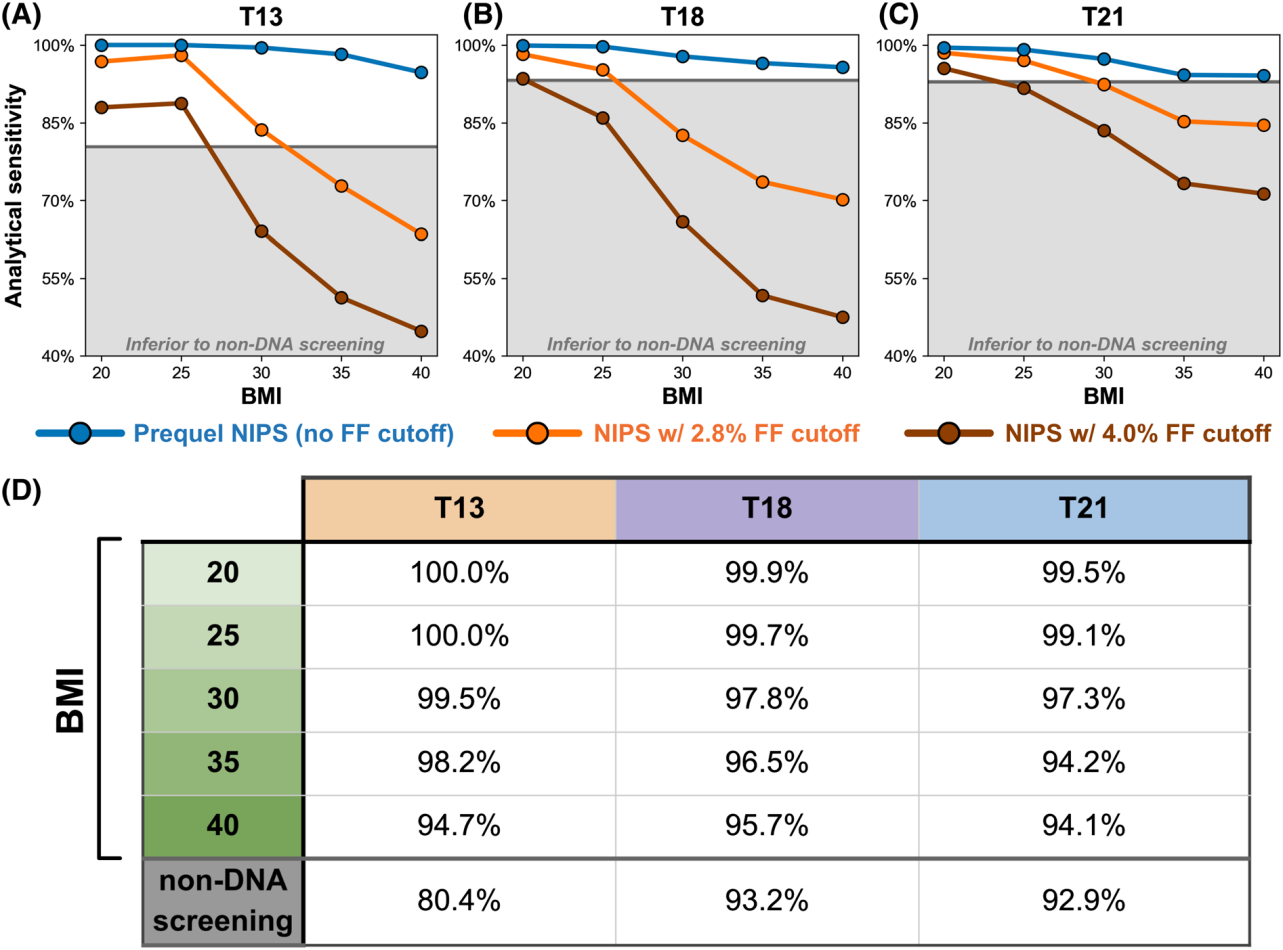
Expected estimated analytical sensitivity for common fetal aneuploidies as a function of maternal body mass index (BMI). A‐C, For T13, T18, and T21, the sensitivity across the entire fetal fraction (FF) distribution is shown as a function of BMI for the Prequel noninvasive prenatal screening (NIPS) without an FF cutoff (blue), the Prequel NIPS if all samples below 2.8% FF were failed (orange), and the Prequel NIPS if all samples below 4.0% FF were failed (brown). The horizontal black line in panels A to C indicates the reported sensitivity of non‐DNA screening (without consideration of BMI), and the gray‐shaded region indicates sensitivities below the non‐DNA screening level. D, The top five rows show expected estimated analytical sensitivity of the indicated aneuploidy and BMI level from blue profiles in panels A to C. The final row indicates the combined sensitivity of non‐DNA screening analysis (without consideration of BMI) [Colour figure can be viewed at http://wileyonlinelibrary.com]

The results of the FF analysis (eg, Figure 3E for BMI = 35) and analytical sensitivity simulations (Figure 4) enabled direct calculation of expected analytical sensitivity for a given aneuploidy and BMI class (Figure 5; see Section 2). For each aneuploidy, even though sensitivity declines as BMI rises, the analytical sensitivity remained above 94%, even for patients with class III obesity (BMI > 40). The estimated analytical sensitivity of our customized NIPS exceeded the clinical sensitivities via non‐DNA screening (blue traces remain above gray region in Figure 5A‐C; Figure 5D).

As a function of BMI, we compared the expected sensitivity of NIPS offerings that fail low‐FF samples to the sensitivity of non‐DNA screening. Aneuploidies among failed samples are undetected by the test and lower a test's actual sensitivity.^26^ As such, we set FF‐specific sensitivity to 0% for FF values below published cutoffs for other implementations of NIPS—2.8% in Ryan et al^8^ and 4% in McCullough et al^9^—and estimated the impact on expected sensitivity as a function of BMI (Figure 5A‐C, orange and brown traces). With either FF threshold, the missed positives among failed samples lowered overall NIPS sensitivity to a level below that of non‐DNA screening. Furthermore, we expect this analysis to have overestimated sensitivity for these tests with FF failure thresholds because they likely have reduced sensitivity approaching the failure threshold, whereas our modeling used idealized sensitivity values shown in Figure 4 for all above‐threshold FF values. In total, for certain NIPS tests that require an FF threshold, the relative sensitivities at high BMI revealed in these data are consistent with the recommendation for using non‐DNA screening instead of NIPS; however, the data also suggest that this recommendation should not be universal because NIPS sensitivity as a function of BMI varies by platform and laboratory.

Though not directly evaluated here, analytical specificity and PPV of WGS‐based NIPS is dictated primarily by the *z*‐score threshold: With the *z*‐score cutoff of 3 in our simulations, the NIPS false‐positive (FP) rate per chromosome was approximately 1 in 1000 or 0.1%. This specificity greatly exceeds that of non‐DNA screening, which was reported to have an overall FP rate of 4.5%.^1^ Together, for women with high BMI, our results suggest that non‐DNA screening has lower sensitivity, specificity, and PPV than our customized WGS‐based NIPS optimized for performance at low FF.

## 4 DISCUSSION

### 4.1 Principal findings and results

The clinical validity and utility for fetal aneuploidy screening is maximized when patients have access to testing with the highest sensitivity, specificity, and PPV. If non‐DNA approaches were strictly superior to cfDNA‐based NIPS on these performance measures among patients with high BMI, then a case could be made for universal recommendation of non‐DNA screening rather than NIPS in that population. However, using a large patient cohort and empirically guided modeling of a customized WGS platform that does not fail samples for having low FF, we have shown that estimated NIPS performance can exceed the clinical performance of non‐DNA screening for patients with high BMI (Figure 5).

### 4.2 Clinical and research implications

BMI‐related test failures are not a benign outcome: They cause patient anxiety and potentially lead to multiple rounds of screening or unnecessary invasive testing,^27^, ^28^ which delays patients' reproductive decision‐making and incurs associated administrative, emotional, and cost burdens. Therefore, taking measures to diminish such test failures is warranted. One approach is to attempt to avoid BMI‐related NIPS test failures altogether by using a screening modality other than NIPS; this is the strategy represented by the recommendation for patients with significant obesity to be offered non‐DNA screening rather than NIPS.^16^ Another potential approach is to mitigate the impact of test failures, eg, using a framework wherein samples that have observed FF significantly below the expected FF are failed and flagged as having elevated risk.^29^ However, the study characterizing this mitigation approach showed that it only applies to a subset of the less‐common aneuploidies (eg, Down syndrome is not included) and has limited utility for patients (those with high BMI are less likely to be flagged as high risk because a significant difference between observed and expected FF is harder to detect when expected FF is itself reduced because of high BMI). A final approach is not to avoid or mitigate BMI‐related NIPS test failures but rather to optimize the NIPS methodology such that the test is effective for patients irrespective of their body shape and size. We have demonstrated that NIPS can be implemented such that it has high sensitivity at low FF and, thus, can serve patients across the BMI spectrum, improving detection of aneuploid pregnancies and diminishing residual risk in euploid pregnancies. Though the recommendation for non‐DNA screening in pregnant women with significant obesity may have merit for NIPS offerings with high test‐failure rates or unexplored efficacy at low FF, providers should be aware of NIPS alternatives that can outperform non‐DNA screening even in patients with very high BMI.

Although much of our analysis focused on low‐FF pregnancies, a striking observation applies to those with normal FF: The majority of women with significant obesity would be underserved by using non‐DNA screening instead of NIPS. Our analysis of FF levels as a function of BMI shows that across all BMI classes—even those with class III obesity—the majority of patients have FF > 4% (Figure 3C). Because a patient's actual FF cannot be known prior to testing, a woman's BMI is only a proxy for FF. However, obese women tested with non‐DNA screening who ultimately would have had normal FF levels receive different and, critically, inferior aneuploidy screening because of the possibility that they might have had low FF.

### 4.3 Strengths and weaknesses

Several caveats should be noted for this study. First, the conclusions rely in part on simulations of WGS‐based NIPS; results may vary on clinical samples and with different algorithms for aneuploidy detection and FF measurement. Unlike a previous study that purposefully compared idealized theoretical implementations of the WGS and SNP methods,^7^ here we aimed to emulate empirical WGS performance of the Prequel NIPS in the simulations: Effective read‐depth, a key input parameter for the simulations, was determined by directly fitting empirical data from our laboratory; thus, our results may not generalize to other laboratories. Second, our calculations are estimates of sensitivity rather than clinical observations of test performance. As we noted earlier, the rarity of low FF and aneuploidy necessitated a mathematically driven analysis. Third, our analyses model the analytical sensitivity of NIPS, whereas the non‐DNA screening comparators are reported clinical sensitivity values. However, the primary driver of the difference between clinical sensitivity and analytical sensitivity is true fetal mosaicism, present in less than 1% of pregnancies with T13, T18, and T21.^30^ Indeed, multiple studies demonstrate the high clinical sensitivity of NIPS.^31^, ^32^, ^33^ Finally, we have assumed non‐DNA screening sensitivity is constant across all BMI classes, but it is well‐known that the ability to obtain an NT measurement decreases with increasing BMI, with some estimates noting failure rates up to 22%.^34^ Therefore, we are likely overestimating the performance of non‐DNA screening in this patient population.

## 5 CONCLUSION

Many pregnant women have high BMI in the United States and have low FF levels that yield elevated test failure rates on most NIPS offerings, highlighting the need for alternatives. Though the alternatives include non‐DNA screening modalities, an NIPS customized and demonstrated to be sensitive at low FF should also be among the alternatives and potentially the preferred option because of its superior sensitivity at all BMI levels.

## FUNDING INFORMATION

Myriad Women's Health (formerly Counsyl) provided funding for all aspects of this study.

## CONFLICT OF INTEREST

D.M., J.D.G., and C.H. are current or former employees of Myriad Genetics.

## Supporting information

Data S1: Supplementary Information

**Figure S1:** For the BMI level indicated in each panel's legend, the best beta‐distribution fit (see Methods) is shown for euploid, T13, T18, and T21 pregnancies.

**Figure S2:** An input to sensitivity simulations is the NGS read‐depth at which the distribution of the number of reads per genomic bin is roughy Poisson. For the four randomly selected clinical samples shown, scaling empirical bin counts by a single number yields distributions well fit by a Poisson distribution (see Methods). Smooth dashed lines are Poisson distributions that were fit to the empirical data, shown as solid jagged traces.

## Data Availability

The data that support the findings of this study are available on request from the corresponding author. The data are not publicly available because of privacy or ethical restrictions.
