# Supplementary material for: Noninvasive prenatal screening for patients with high body mass index: Evaluating the impact of a customized whole genome sequencing workflow on sensitivity and residual risk
Source: Prenat Diagn. 2019 Dec 20;40(3):333–41. doi: 10.1002/pd.5603 (PMC7065115; doi:10.1002/pd.5603)
Supplement: Supplementary file 1 — Data S1: Supplementary Information [file PD-40-333-s001.docx]

## SUPPLEMENTAL METHODS

## Noninvasive prenatal screening for patients with high body mass index: Evaluating the impact of a customized whole genome sequencing workflow on sensitivity and residual risk

Dale MUZZEY^1,2^, PhD, James D. GOLDBERG^1^, MD, and Carrie HAVERTY^1^, MS CGC

^1^Myriad Women’s Health, 180 Kimball Way, South San Francisco, CA, 94080

^2^Myriad Genetics, 320 Wakara Way, Salt Lake City, UT, 84108

####

#### Empirically informed WGS simulation to measure sensitivity as function of FF

One batch (n=114 samples) of Prequel clinical samples with typical sequencing metrics was selected at random. For each sample, the number of next generation sequencing (NGS) reads aligning to each nonoverlapping 20kb bin tiling the genome was adjusted to account for factors like GC bias and repetitive sequence elements that could cause systematic non-uniformity in the distribution of observed reads per bin [^1^](https://paperpile.com/c/5pbuEU/LxWDp).

For each sample, adjusted bin values from autosomes were aggregated into histograms, and goodness of fit was assessed with respect to a Poisson distribution via the sum-squared difference between the empirical data and theoretical fit. Empirical bin values were scaled to yield a best fit to the Poisson distribution and then summed to provide a total number of NGS reads at which the test can be modeled as a Poisson process.

Across many samples, the median of adjusted-bin-value sums was calculated and served as the input to previously described simulations of WGS-based NIPS [^2^](https://paperpile.com/c/5pbuEU/i9TbQ). The simulation software assumed Poisson sampling by default, modeled data for aneuploid samples, assigned a z-score to each chromosome describing its deviation from an expected euploid baseline, and used the following parameters: FF, aneuploid region (T13, T18, or T21), and the total number of reads devoted to a sample. FF-dependent analytical sensitivity was calculated as the fraction of simulated positive samples that had *z* > 3.

#### Fetal-fraction distributions as function of BMI and aneuploidy

For samples with male fetuses, FF was based on the average NGS-read density on allosomes. For samples with female fetuses, FF was calculated via a ridge-regression model trained on the mapping of autosomal bin counts onto allosomal-derived FF in males [^3^](https://paperpile.com/c/5pbuEU/tnNPc).

To represent FF as a continuous and parametric function with effectively infinite resolution at low FF (where empirical samples are rare), we fit a beta distribution to the FF data for each BMI class (see Results).

To model an FF distribution that simultaneously represents a particular BMI class and aneuploidy (e.g., the FF distribution of T13 pregnancies in patients with class III BMI), we had to overcome the limitation that positive samples are relatively rare after partitioning them into BMI classes: fitting a beta distribution to such sparse empirical data may not accurately represent the underlying truth. Therefore, we derived such a distribution indirectly by leveraging deeply sampled portions of the dataset (e.g., screen-negative across the whole BMI spectrum). Specifically, for a given aneuploidy, within each BMI class we partitioned samples into screen-positive and screen-negative pools. For each screen-positive sample, we determined its percentile relative to the screen-negative population. All such percentile scores were aggregated across BMI classes into a single “master list” of percentiles. To build the expected distribution of aneuploid samples in a given BMI class, FF values were selected from the screen-negative distribution for that BMI class according to the percentiles in the master list. Finally, a beta distribution was fit to the resulting list of FF values. In summary, this process leveraged both the relative abundance of screen-negative samples in each BMI class and the general FF skew in screen-positive calls across BMI classes to enable principled fitting of an FF distribution.

1. [Muzzey D. The Technology and Bioinformatics of Cell-free DNA based NIPT. In: Lieve Page-Christiaens H-GK, ed. *Noninvasive Prenatal Testing (NIPT) Applied Genomics in Prenatal Screening and Diagnosis*. Elsevier; 2018.](http://paperpile.com/b/5pbuEU/LxWDp)

2. [Artieri CG, Haverty C, Evans EA, et al. Noninvasive prenatal screening at low fetal fraction: comparing whole-genome sequencing and single-nucleotide polymorphism methods. *Prenat Diagn*. 2017;37(5):482-490.](http://paperpile.com/b/5pbuEU/i9TbQ)

3. [Kim SK, Hannum G, Geis J, et al. Determination of fetal DNA fraction from the plasma of pregnant women using sequence read counts. *Prenat Diagn*. 2015;35(8):810-815.](http://paperpile.com/b/5pbuEU/tnNPc)
