# Supplementary figures and images for: Noninvasive prenatal screening for patients with high body mass index: Evaluating the impact of a customized whole genome sequencing workflow on sensitivity and residual risk
Source: Prenat Diagn. 2019 Dec 20;40(3):333–41. doi: 10.1002/pd.5603 (PMC7065115; doi:10.1002/pd.5603)

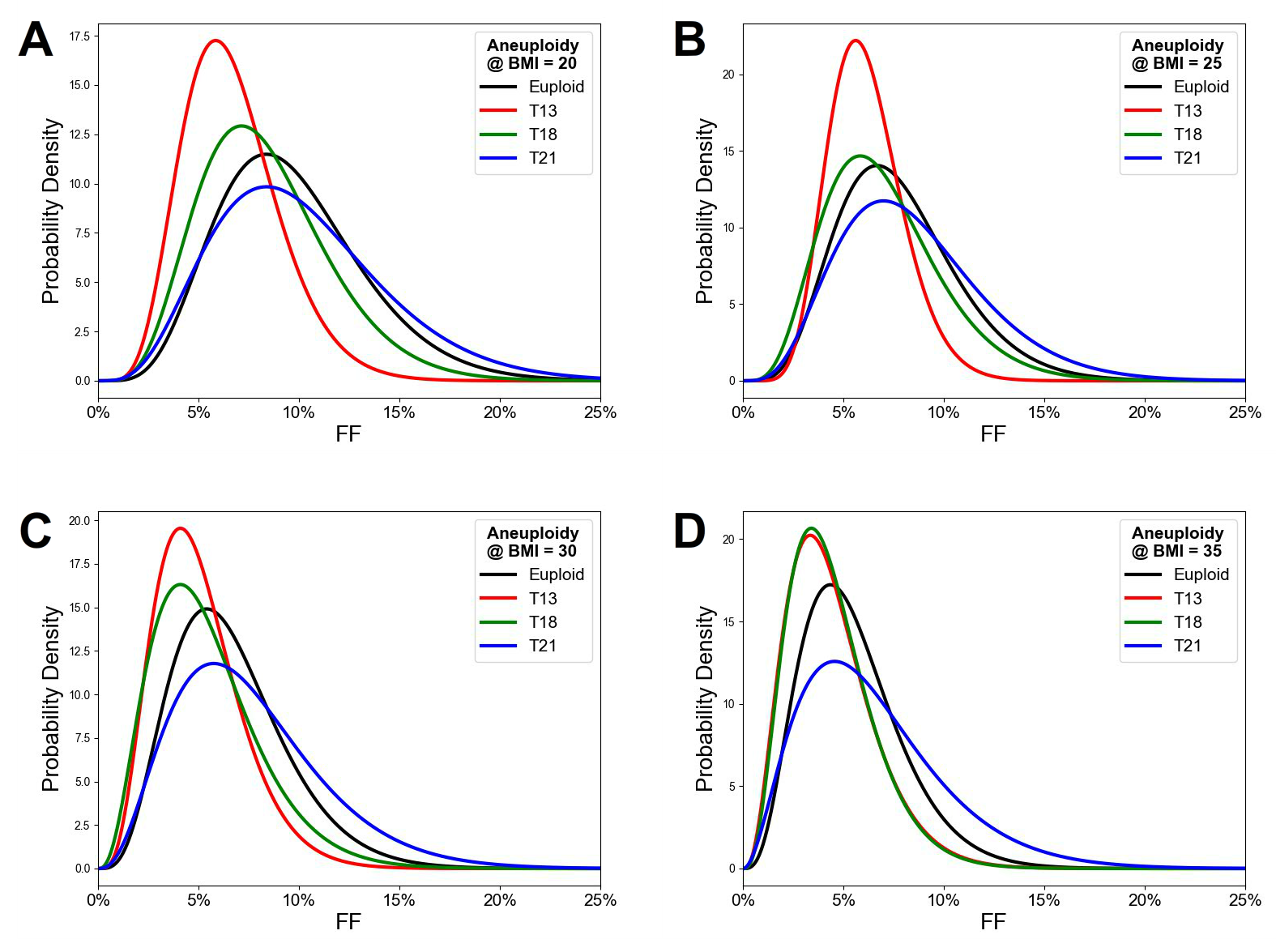

Supplement: Supplementary file 2 — Figure S1: For the BMI level indicated in each panel's legend, the best beta‐distribution fit (see Methods) is shown for euploid, T13, T18, and T21 pregnancies. [file PD-40-333-s002.png]

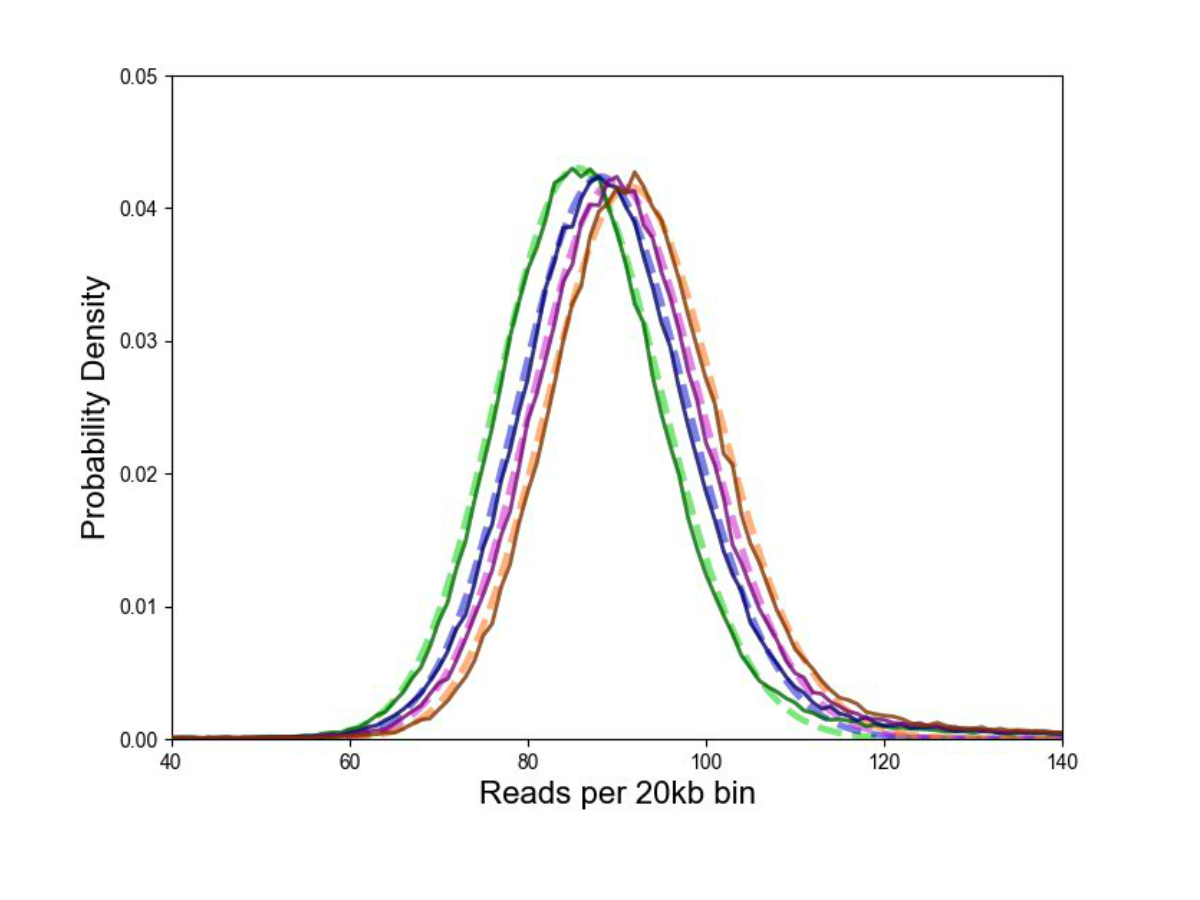

Supplement: Supplementary file 3 — Figure S2: An input to sensitivity simulations is the NGS read‐depth at which the distribution of the number of reads per genomic bin is roughy Poisson. For the four randomly selected clinical samples shown, scaling empirical bin counts by a single number yields distributions well fit by a Poisson distribution (see Methods). Smooth dashed lines are Poisson distributions that were fit to the empirical data, shown as solid jagged traces. [file PD-40-333-s003.png]
